# Aboveground plant-to-plant communication reduces root nodule symbiosis and soil nutrient concentrations

**DOI:** 10.1038/s41598-021-92123-0

**Published:** 2021-06-16

**Authors:** Yuta Takahashi, Kaori Shiojiri, Akira Yamawo

**Affiliations:** 1 Department of Biology, Faculty of Agriculture and Life Science, Hirosaki University, 1 Bunkyo-cho, Hirosaki, 036-8560 Japan; 2 Faculty of Agriculture, Ryukoku University, Otsu, Shiga 520-2194 Japan

**Keywords:** Biochemistry, Ecology, Plant sciences, Ecology, Environmental sciences, Planetary science

## Abstract

Aboveground communication between plants is well known to change defense traits in leaves, but its effects on belowground plant traits and soil characteristics have not been elucidated. We hypothesized that aboveground plant-to-plant communication reduces root nodule symbiosis via induction of bactericidal chemical defense substances and changes the soil nutrient environment. Soybean plants were exposed to the volatile organic compounds (VOCs) from damaged shoots of *Solidago canadensi*s var. *scabra*, and leaf defense traits (total phenolics, saponins), root saponins, and root nodule symbiosis traits (number and biomass of root nodules) were measured. Soil C/N ratios and mineral concentrations were also measured to estimate the effects of resource uptake by the plants. We found that total phenolics were not affected. However, plants that received VOCs had higher saponin concentrations in both leaves and roots, and fewer root nodules than untreated plants. Although the concentrations of soil minerals did not differ between treatments, soil C/N ratio was significantly higher in the soil of communicated plants. Thus, the aboveground plant-to-plant communication led to reductions in root nodule symbiosis and soil nutrient concentrations. Our results suggest that there are broader effects of induced chemical defenses in aboveground plant organs upon belowground microbial interactions and soil nutrients, and emphasize that plant response based on plant-to-plant communications are a bridge between above- and below-ground ecosystems.

## Introduction

Several studies have pointed out that physiological integration of roots and shoots in plants has altered our understanding of aboveground–belowground interactions^1–3^. Many studies demonstrated that herbivory in belowground parts affects the expression of defense traits in aboveground tissues^1–3^. However, a comparatively few studies have provided evidence of the aboveground leaf damage responses influencing root traits or -associated organisms^1^. For example, foliar application of jasmonic acid to grape vines reduced the number of root-feeding grape phylloxera to about half the numbers on control plants^4^. Leaf damage on veins suppresses root foraging precision^5^.

Plant-to-plant communications mediated by volatile organic compounds (VOCs) have been studied in many plant species^6,7^. Damaged plants release specific VOCs such as methyl jasmonate and salicylate. These VOCs from herbivore-damaged plants activate the expression of resistance genes, prime resistance in surrounding undamaged plants^6–8^, and alter the behavior of herbivores^9^, thus reducing future herbivory in undamaged plants^9–11^. Through these processes, plant-to-plant communication imparts adaptability to undamaged plants.

Plant-to-plant communication is also mediated by VOCs from artificially damaged plants^6–8^. Sagebrush plants (*Artemisia tridentata*) exposed to VOCs from artificially damaged conspecifics, suffer less herbivory than those exposed to volatiles from undamaged ones^12^. Similarly, young undamaged seedlings of *Chrysanthemum cinerariaefolium* exposed to volatiles from artificially damaged conspecifics increase their content of pyrethrin, a chemical defense substance^13^. Plant-to-plant communication mediated by VOCs has also been reported between artificially damaged heterospecific plants^11,14,15^. For example, VOCs from goldenrod *Solidago canadensis* increased the defenses of soybean plants against herbivores^11^ through changes in chemical traits, saponin concentrations, or total phenolics^16^. The plant-to-plant communications among heterospecific plants were considered to be adaptive against generalist herbivores. Thus, plant-to-plant communications play an important role in plant–herbivore interactions in aboveground organs; however, the effects of plant-to-plant communication on biotic interactions in belowground plant organs and soil environments with regards to soil nutrient levels have not been reported.

Plastic changes that are induced by plant-to-plant communication could affect roots through byproducts of physiological mechanisms. Plant-to-plant communications often lead to increases in the content of secondary chemical metabolites in leaves^7–16^ or changes in shoot-to-root allocation^17^. The increase in secondary chemical metabolites may affect the microbial community associated with roots because secondary chemical metabolites have anti-microbial properties^18–21^. For example, Toth et al.^20^ reported that susceptible maize inbred lines had significantly higher levels of mycorrhizal colonization than resistant inbred lines. Non-mycorrhizal plants tend to have higher levels of chemical defenses^22^.

The formation of symbiotic root nodules is one type of plant–microbe interaction, through which primarily leguminous species obtain nitrogen fixed by nitrogen-fixing rhizobia. To drive this symbiotic process, plants allocate carbon that could have been used for their growth, reproduction, and defenses to the symbiotic microbes in exchange for nitrogen^23,24^. Therefore, resource allocation from shoot to root in response to plant communication may reduce the root nodule symbiosis. Moreover, in the Fabaceae family, plants produce chemical metabolites such as phenols and saponins that induce anti-herbivore defense after when they receiving VOCs released from damaged plants^25,26^. Thus, we hypothesized that plant-to-plant communication would reduce root nodule symbiosis traits through byproducts of induced defense or a change in shoot-to-root allocation^17,20–22^.

Moreover, the change in root nodule symbiosis may influence the soil mineral concentrations through alterations in the resource uptake by the plants. Plants modify the resource uptake in response to various environmental conditions. For example, limiting root nodule symbiosis by soil sterilization increases root allocation and soil nutrient uptake^20^. If root nodule symbiosis is inhibited by induced defense, plants require increased nutrient uptake^20^. In such a case, soil nutrients around the plants that received VOCs are hypothesized to be less abundant compared with those of plants not exposed to VOCs.

To test these hypotheses, we conducted a greenhouse study using soybean, *Glycine max*, as the assay plant and goldenrod, *Solidago canadensis*, as the emitter plant. This combination has been used to study plant-to-plant communications, and defenses in soybean plants have been shown to be induced by exposure to the VOCs from goldenrod^11^. We grew soybean plants in the presence of the emitter plant (plant-to-plant communication) and in its absence (control) conditions in the greenhouse, and after 3 weeks of growth, measured leaf and/or root defense traits (total phenolic compounds and saponin concentrations), plant biomass, number and biomass of root nodules, and soil nutrient and minerals contents.

## Materials and methods

### Plant cultivation

Seventy seeds of Soybeans, *Glycine max*, were sown on the surface of moist soil (2 cm deep) in plastic containers (7 cm wide × 9 cm long × 5 cm deep) in June 2020. The containers were maintained in a growth chamber at 25 °C under a 12/12 h light/dark photoperiod. Water was provided every second day (300 mL). After 15 days, 70 germinated plants were individually planted in plastic pots (5 × 5 × 10 cm) containing garden soil (Sun and Hope Co., Tokyo, Japan). The pots were maintained in a growth chamber at 25 °C under a 12/12 h light/dark photoperiod for 10 days. The experiment commenced when the plants reached the three- or four-leaf stage, because plant defenses for future growth are often developed at the seedling stage^27^.

### Experimental design

The plants were randomly assigned to either the control (no exposure to VOCs, *n* = 35) or the communication treatment group (exposed to VOCs, *n* = 35, Fig. S1) . To prevent the control plants from receiving VOCs, the two groups were placed in different rooms (5 × 20 × 4 m) in a greenhouse separated by a glass wall at Hirosaki University (40°59ʹN, 140°47ʹE) in north Japan. Both rooms were maintained at 25 °C with natural light conditions. In the room with the communication treatment, five 3 mm mesh bags (25 × 25 cm) containing cut goldenrod shoots (10–20 cm pieces, 300 g) collected at Hirosaki University just before the experiment were placed at 60 cm intervals. These goldenrods had less than 1% leaf damage, no flowers, and belonged to two large patches of goldenrod more than 30 m apart. The cut goldenrod treatment induces production of artificial damage-dependent VOCs^11^. Seven potted soybean plants were arranged around each mesh bag within 30 cm, with the mesh bags replaced every 5 days in accordance with a previous study^11^. In the other room, five empty mesh bags were used for the control treatment. After 3 weeks, the soybean plants were harvested and dried at 40 °C for 3 days. Then the plants were divided into roots, shoots, and root nodules and weighed on an electronic balance. The number of root nodules on each plant was recorded.

### Leaf traits

The contents of phenolic compounds and condensed tannins in leaves were measured according to Feeny^28^ and Dudt and Shure^29^. Dried plant tissues were powdered in a mill. Total phenolics were extracted from leaf powder (20 mg) in 50% methanol (10 mL) for 1 h in an ultrasound bath at 40 °C. The Folin–Ciocalteu method was used to measure the phenolic compound concentration (mg g^–1^)^30,31^. Phenolic compounds were determined according to the reduction of Mo^6+^ to Mo^5+^, which is blue and can be measured optically at 730 nm^32^.

The content of saponins was measured according to the studies by Mukai et al.^33^ and Dubois et al.^34,35^. Dried plant tissues were powdered in mill. Total saponins were extracted from plant powder (20 mg) in 80% methanol (40 mL) for 12 h at 25 °C. The phenol–sulfuric acid method which involves the coloration of the sugar chain of saponin was used to measure saponin concentration (mg g^–1^).

### Soil nutrients and minerals

After the experiment, soil samples (approximately 1000 mg) were collected from each pot, and the samples were air-dried, mixed, and the stones were removed. Carbon and nitrogen contents of the soil were measured using an elemental analyzer (Vario MAX cube, Elementar), with 500–600 mg samples compared between the experimental conditions. Because the same soil was used in both the control and communicated treatment groups, we used C/N ratio as an indicator of the soil nutrient status. Moreover, 14 exchangeable soil minerals, Cu, Si, Li, Na, P, K, Ca, B, Mg, S, Mo, Fe, Mn, and Zn, were extracted from 1 g air-dried soil using 1 M ammonium acetate (pH 7.0)^36^ and measured by inductively coupled plasma atomic emission spectroscopy (ICP-AES) (iCAP7000 Series; Thermo Scientific, Wilmington, DE, USA).

### Data analysis

All statistical analyses were performed in R v. 4.0.2 software^37^. Plant biomass, total phenolics, saponins, dry weight of root nodules, and soil C/N ratio were compared between the treatments in linear models. Number of root nodules was compared in generalized linear models (GLMs) with a Poisson distribution and log link. These models included the experimental treatment as the explanatory variable. Since root nodule biomass depends on aboveground biomass, aboveground biomass was included as a covariate in the models for the number and dry weight of root nodules. The *P*-values were corrected by the false discovery rate. Saponin content from the leaf and root was compared using the generalized linear mixed model with Gaussian distribution and identity link. Plant parts (leaf or root) were included as fixed effects, and treatments (control or communicated) were included as random effects in the model. The relationships between saponins and number or dry weight of root nodules were analyzed by GLMs with a Gaussian distribution and identity link. Condensed tannins were removed from the analysis because it was not detected. Soil minerals were analyzed by a principal component analysis (PCA) based on the correlation matrix of variables. Scores on first (PC1) and second axis (PC2) of PCA were compered between experimental conditions using GLM.

### Ethics approval

The experimental research and field studies on plants, including the collection of plant material, complied with relevant institutional, national, and international guidelines and legislation. The appropriate permissions and/or licenses for collection of plant or seed specimens were obtained for the study.

## Results and discussion

**Figure 1:**
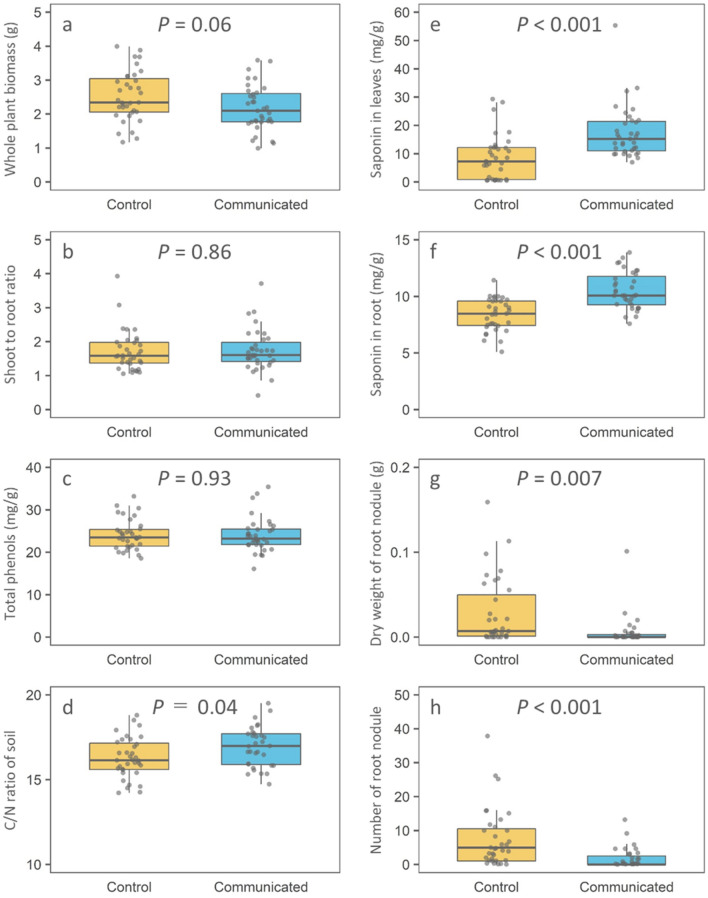
Growth, defense traits and root nodule symbiosis in soybean and soil nutrient concentration. (**a**) Whole plant biomass (g), (**b**) Shoot to root ratio, (**c**) total phenolics (mg g^–1^), (**d**) C/N ratio in soil, (**e**) saponin concentration in leaves (mg g^–1^), (**f**) saponin concentration in root (mg g^–1^), (**g**) dry weight of root nodules per plant (g), (**h**) number of root nodules per plant. Data are means ± SD (control, n = 35; communicated treatment plants, n = 35). *P*-values were corrected by the false discovery rate. Boxplots were plotted in R using the ggplot2 package.

Similar to many previous studies^6,7,11^, our experiment demonstrated that the plant-to-plant communication induced changes in plant quality. Although plant biomass, shoot-to-root ratio and total phenolics in leaves were not affected (Fig. 1a–c), the saponins in both leaves and roots were significantly higher in the VOC-exposed plants (Fig. 1e,f). Saponins are well known as defense substances against various herbivore species including *Spodoptera litura* that are major herbivores of soybean^26,38^. Our results are consistent with those of Shiojiri et al.^11^ who showed that the VOCs from cut goldenrod increase the defense of soybean plants against the herbivore, *S. litura*. The response of soybean plants may reduce leaf damage through resistance effects^6–8^ or alteration of herbivore behavior^9^.

Notably, our findings strongly suggest that plant-to-plant communication affected root nodule symbiosis. The number and dry weight of root nodules were significantly smaller in communication plants than in control plants (Fig. 1g,h). One of the possible causes of the reduction in root nodules is the change in carbon allocation in response to plant-to-plant communication. However, we could not find differences in resource allocation between control and communicated plants (Fig. 1b). In contrast, we found negative correlations between saponin concentrations and root nodule number and biomass (number of root nodules, estimate coefficient = − 0.01, *F* = 3.45, *P* < 0.001; dry weight of root nodule, which is estimate coefficient = − 2.26, *F* = 3.35, *P* < 0.001). Although we did not evaluate the N fixing activity of nodules, one of the major factors of N accumulation^39^, our results indicate that it at least affects the frequency of root nodule symbiosis. One explanation is that saponin is a byproduct of induced defenses, as we hypothesized. Compared with roots, higher content of saponins in leaves (*F* = 11.23, *P* = 0.001) indicated that leaves could be the main organs of saponin synthesis or storage, regardless of the plant-to-plant communication status. Saponins are known to have antibacterial functions^40^. These results strongly suggest that increased saponin concentrations in communicated plants inhibited root nodule symbiosis. Thus, the cost of plant-to-plant communication in soybean is to prevent the establishment of root nodule symbiosis in plants.

Saponins are a vast group of glycosides that have a wide antimicrobial activity and are widely distributed in higher plants^40–42^. 5β-Spirostan-3β-ol saponins have antimicrobial activity in both prokaryotic and eukaryotic organisms^43^. Therefore, the induction of saponins in response to VOCs in plants may affect not only the root nodule symbiosis, but also other interactions between microbial organisms and plants such as mycorrhizal symbiosis. The effects of chemical defense on the interactions between microbial organisms and plants may also be found in macroevolutionary relationships between chemical defense and microbial symbiosis. A few studies have revealed that plants that have non-mycorrhizal or low levels of mycorrhizal colonization tend to have high levels of chemical defenses^20,22^. Our results also support the conventional idea that plant secondary chemicals shape plant–microbe interactions^e.g.^^20,22,44^.

The negative effects of chemical defenses on mutualisms have been reported in plant–pollinator mutualism. The induction of plant defense against herbivory increases the concentration of defensive chemical compounds in the nectar of flowers, and this reduces pollinator attraction and seed production^45^. These results suggest the negative effects of chemical defense substance on mutualistic interactions. Future studies should focus on the relationships between variations in chemical defense and mutualistic interactions to understand variations in these mutualistic interactions.

How does the release of rhizome symbiosis affect the use of soil nutrients by plants? Our soil resource analysis revealed lower concentrations of resources in soil around communicated plants than that in soil around the control plants (Fig. 1d). PC1 and PC2 explained 96.2% and 2.4% of the total variance of soil mineral composition data, respectively. Soil mineral composition did not differ between experimental soils (PC1, *F* = 0.31, *P* = 0.76; PC2, *F* = 0.19, *P* = 0.85; Fig.S2). Thus, plant-to-plant communication influenced the soil nutrient status through changes in root-nodule symbiosis and the rate of plant nutrient uptake. In other words, the reduction in root nodule symbiosis due to plant-to-plant communication reduces the soil nutrient content through increased nutrient uptake by plants. The effects of plant-to-plant communication on soil status may affect other ecological components such as the plant and microbial community, because soil nutrient status is one of the major drivers of plant^46^ and microbial communities^47^.

In conclusion, we present experimental evidence that aboveground plant-to-plant communication treatment increases saponin concentration and reduces root nodule symbiosis. The findings suggest that the induction of chemical defense has a negative effect on root nodule symbiosis. Moreover, the reduction in root nodule symbiosis decreases soil nutrient concentration. Previous studies on aboveground plant-to-plant communication focused on changes in aboveground plant traits and associated communities^48,49^. Our results elucidate that plant-to-plant communication changes not only the aboveground biological community, but also the belowground bacterial symbiosis and soil environments. The effects of plant ontogeny and plant-to-plant communications, such as intraspecific or interspecific communications, is an interesting avenue for future research, because these factors often produce varying outcomes of plant-to-plant communications. From these perspectives, future studies on the effects of plant-to-plant communication may reveal broader effects on belowground plant tissues.

## Supplementary Information

Supplementary Information 1.

Supplementary Information 2.
