# Supplementary material for: Aboveground plant-to-plant communication reduces root nodule symbiosis and soil nutrient concentrations
Source: Sci Rep. 2021 Jun 16;11:12675. doi: 10.1038/s41598-021-92123-0 (PMC8209107; doi:10.1038/s41598-021-92123-0)
Supplement: Supplementary file 1 — Supplementary Information 1. [file 41598_2021_92123_MOESM1_ESM.docx]

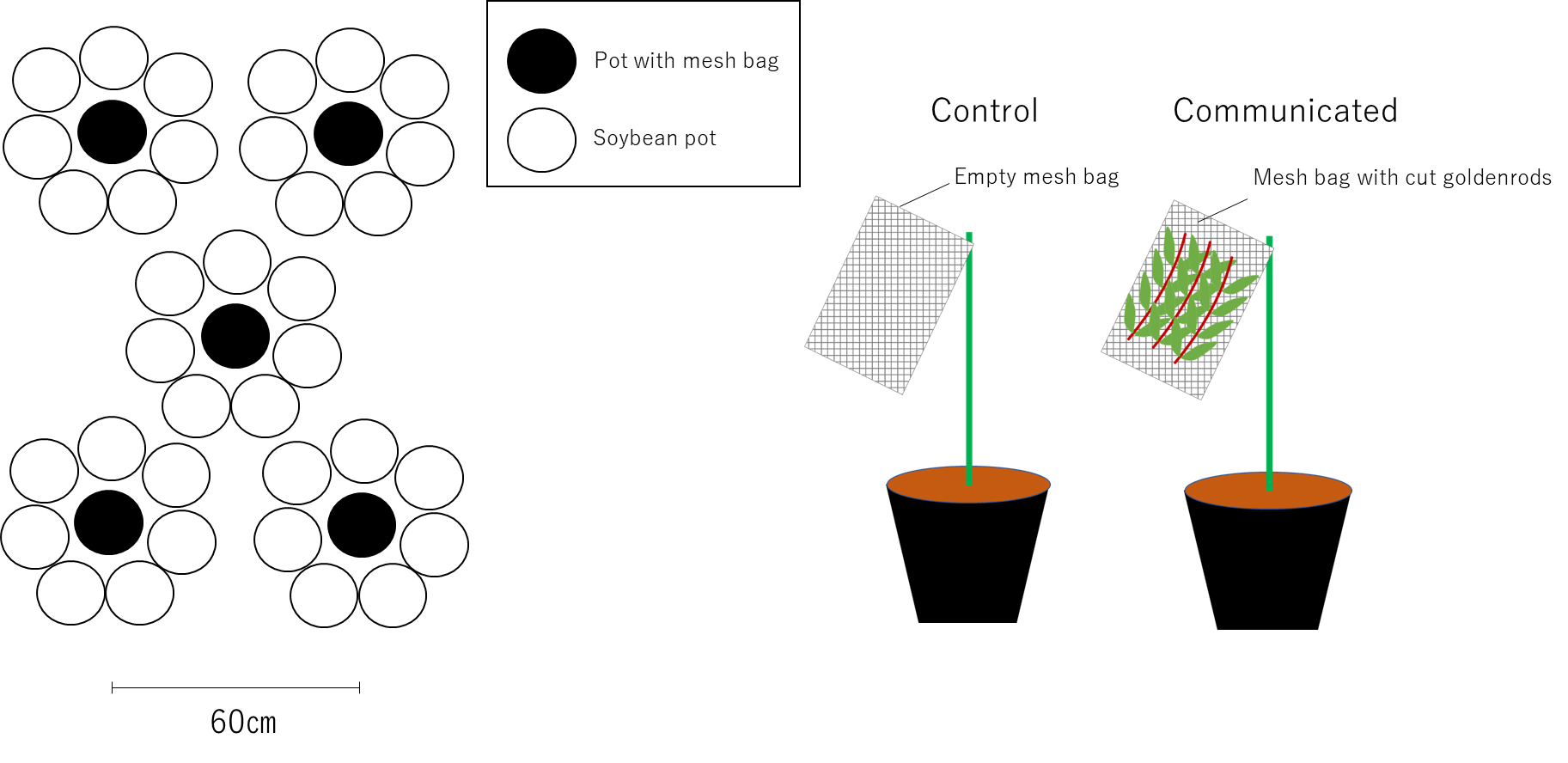


**Figure S1.** Experimental setup for plant-to-plant communication treatment. Mesh bags filled with cut goldenrods were placed in communicated treatment group and empty mesh bags were placed in the control. Images were made in PPT by Yamawo.


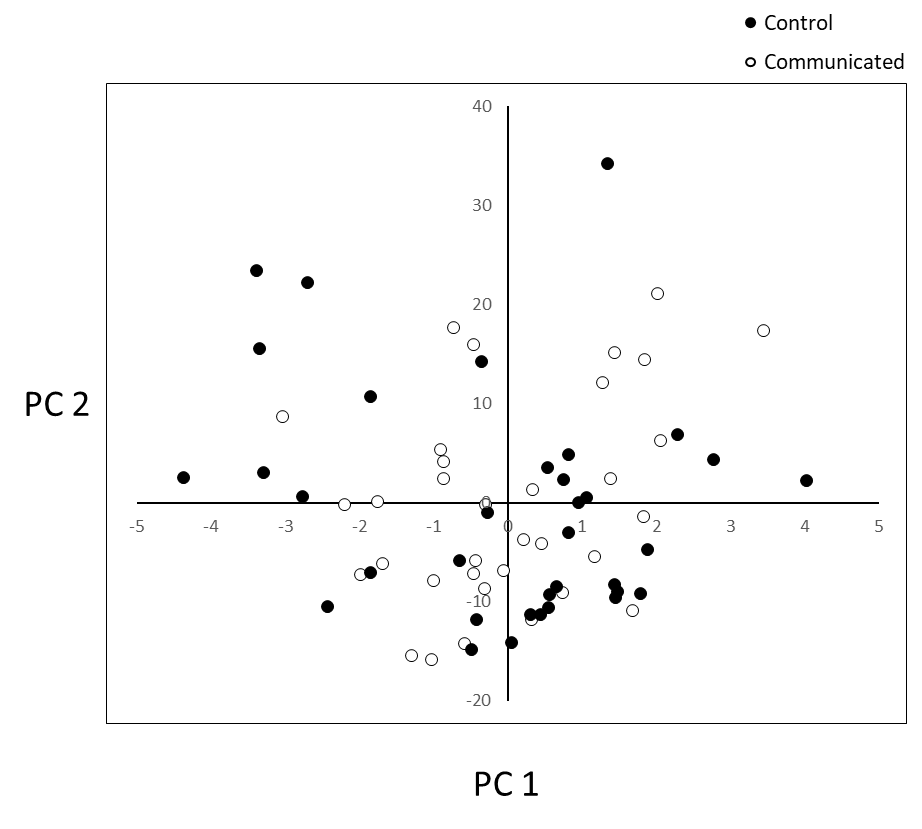


**Figure S2.** Principal component analysis of 14 soil minerals. Scatter plot were plotted in R.
