# Supplementary material for: Aboveground plant-to-plant communication reduces root nodule symbiosis and soil nutrient concentrations
Source: Sci Rep. 2021 Jun 16;11:12675. doi: 10.1038/s41598-021-92123-0 (PMC8209107; doi:10.1038/s41598-021-92123-0)
Supplement: Supplementary file 2 — Supplementary Information 2. [file 41598_2021_92123_MOESM2_ESM.doc]

Table S1 Statistical results of general linear model examining the effects of communicate treatment on whole plant biomass, shoot to root ratio, total phenolics, C/N ratio in soil, saponin in leaves or root, biomass or number of root nodule.

| Factors | df | *F*-value | *P*-value |
| --- | --- | --- | --- |
| Whole plant biomass | 1 | 4.32 | 0.06 |
| Shoot to root ratio | 1 | 0.03 | 0.86 |
| Total phenolics | 1 | 0.007 | 0.93 |
| C/N ratio in soil | 1 | **5.15** | **0.04** |
| Saponin in leaves | 1 | **17.61** | **< 0.001** |
| Saponin in root | 1 | **33.27** | **< 0.001** |
| Biomass of root nodule | 1 | **9.13** | **0.007** |
| Number of root nodule | 1 | **438.66** | **< 0.001** |

**Bold** indicates *P* < 0.05.
